# Analysis of research hotspots and trends in pediatric ophthalmopathy based on 10 years of WoSCC literature

**DOI:** 10.3389/fped.2024.1405110

**Published:** 2024-05-30

**Authors:** Qianfang Jia, Xiaofang Wang, Xiwan Li, Cuijuan Xie, Qing Zhang, Jingfeng Mu, Weihua Yang

**Affiliations:** ^1^ Department of Children Rehabilitation, First Affiliated Hospital of Xinxiang Medical University, Xinxiang, China; ^2^ Xinxiang Autism Integration Education Engineering Technology Research Center, Xinxiang, China; ^3^ Hebei University of Chinese Medicine, Shijiazhuang, China; ^4^ Shenzhen Eye Institute, Shenzhen Eye Hospital, Jinan University, Shenzhen, China; ^5^ The Third Affiliated Hospital of Xinxiang Medical University, Xinxiang Medical University, Xinxiang, China

**Keywords:** pediatric, ophthalmopathy, bibliometric, visualization, CiteSpace, hotspots, trends

## Abstract

**Background:**

Ophthalmopathy occurring in childhood can easily lead to irreversible visual impairment, and therefore a great deal of clinical and fundamental researches have been conducted in pediatric ophthalmopathy. However, a few studies have been performed to analyze such large amounts of research using bibliometric methods. This study intended to apply bibliometric methods to analyze the research hotspots and trends in pediatric ophthalmopathy, providing a basis for clinical practice and scientific research to improve children's eye health.

**Methods:**

Publications related to pediatric ophthalmopathy were searched and identified in the Web of Science Core Collection (WoSCC) database. Bibliometric and visualized analysis was performed using the WoSCC analysis system and CiteSpace.6.2.6 software, and high-impact publications were analyzed.

**Results:**

This study included a total of 7,177 publications from 162 countries and regions. Of these, 2,269 from the United States and 1,298 from China. The centrality and H-index were highest in the United States at 0.27 and 66, respectively. The University of London and Harvard University had the highest H-index at 37. Freedman,Sharon F published 55 publications, with the highest H-index at 19. The emerging burst keyword in 2020–2023 was “eye tracking,” and the burst keywords in 2021–2023 were “choroidal thickness,” “pediatric ophthalmology,” “impact” and “childhood glaucoma.” Retinopathy of prematurity, myopia, retinoblastoma and uveitis in juvenile idiopathic arthritis were the main topics in the high-impact publications, with clinical studies in the majority, especially in retinopathy of prematurity.

**Conclusion:**

Eye health in children is a research hotspot, with the United States publishing the largest number of papers and having the greatest influence in research on pediatric ophthalmopathy, and China coming in second. The University of London and Stanford University had the greatest influence. Freedman, Sharon F was the most influential author. Furthermore, “choroidal thickness,” “pediatric ophthalmology,” “impact,” “childhood glaucoma” and “eye tracking”are the latest hotspots in the field of pediatric ophthalmopathy. These hotspots represent hot diseases, hot technologies and holistic concepts, which are exactly the research trends in the field of pediatric ophthalmopathy, providing guidance and grounds for clinical practice and scientific research on children's eye health.

## 1 Introduction

Ophthalmopathy including include myopia, retinopathy, and glaucoma during childhood can significantly impact the development of visual, potentially leading to irreversible visual impairment, seriously affecting children's health and even contributing to long-term adverse social consequences. Specifically, myopia, in particular may affect children's quality of life and even posing a risk for blindness (1–3). Retinopathy of prematurity, a vasculopathy affecting the developing retinal vasculature in premature newborns, can result in retinal detachment, visual impairment, and blindness (4, 5). Glaucoma, which, if untreated timely, can cause sustained intraocular pressure increases, leading to optic nerve damage and vision loss (6, 7). Thus, timely diagnosis and treatment of ocular diseases in children are crucial, and modern technologies are required to apply in pediatric ophthalmopathy (8, 9). A holistic understanding of the current status and hotspots of research in this area can better provide reference for clinical workers and directions for researchers in pediatric ophthalmopathy.

Bibliometric analysis, first introduced in 1987, has been extensively applied across various fields to objectively assess research dynamics and frontiers, uncover available information in specific areas, and explore future research directions (10–12). While earlier studies have employed bibliometric methods to analyze pediatric ophthalmopathy, they have primarily focused on individual diseases, such as glaucoma (13), retinopathy of prematurity (14), and retinoblastoma (15), failing to offer a comprehensive overview and evaluation of pediatric ophthalmopathy. Consequently, this study conducted a bibliometric and visualized analysis of pediatric ophthalmopathy research to explore the research hotspots and trends in pediatric ophthalmopathy. Additionally, the Web of Science Core Collection (WoSCC) is the standard data set underpinning the journal impact metrics found in the Journal Citation Reports, providing high quality literature (16, 17); and the last decade literature reflects the latest research hotspots and trends that are more valuable to researchers as a research guide. Therefore, the data of this study was based on the literature of Woscc for the last decade to explore the research hotspots and trends in pediatric ophthalmopathy, potentially guiding future research in this field.

## 2 Materials and methods

**Figure 1:**
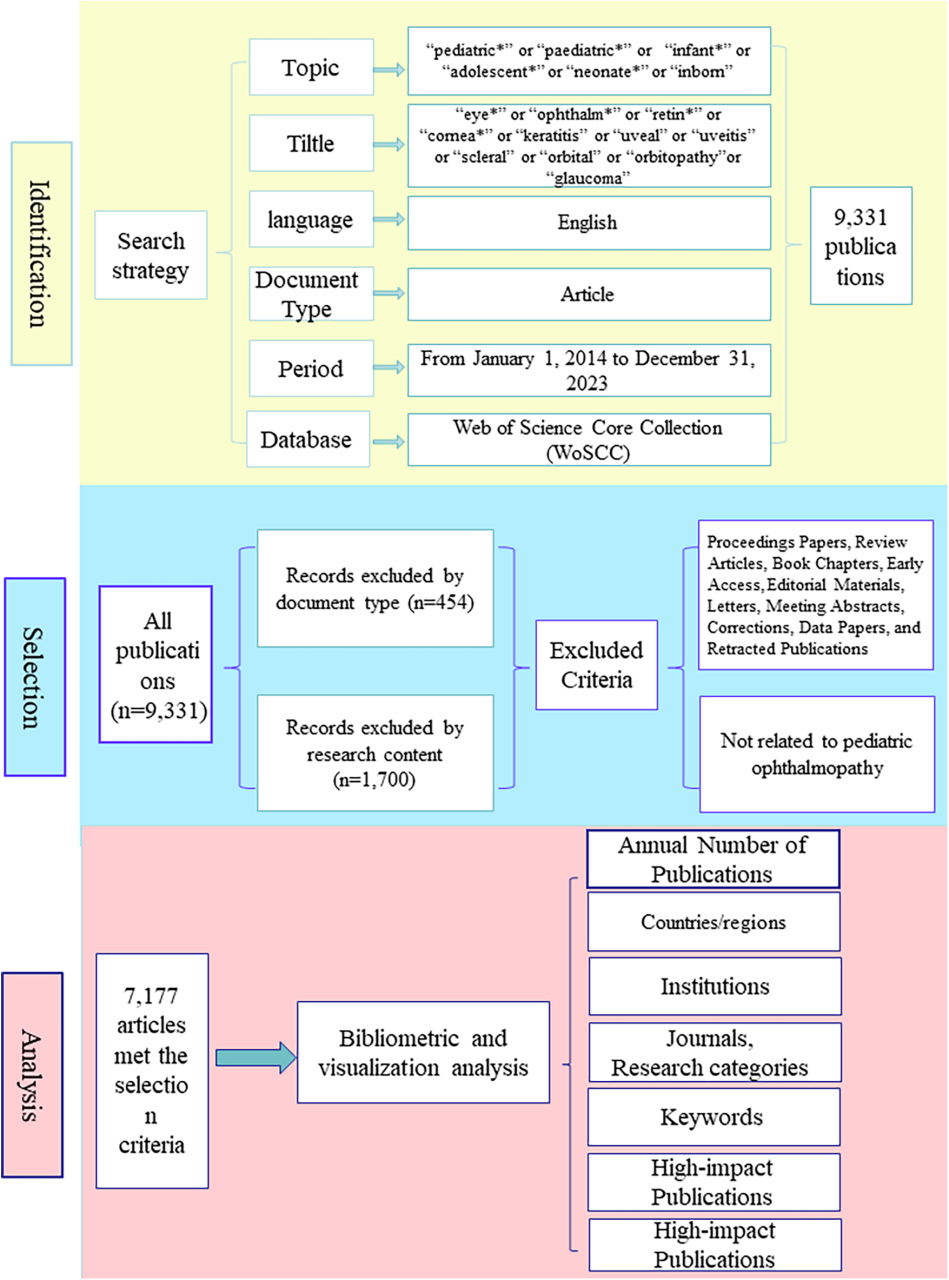
Frame flow diagram showing the detailed selection criteria and bibliometric analysis steps for the study of pediatric ophthalmopathy.

The research methods included three parts: identifying the search strategy, selecting the publications and analyzing the data. In efforts to demonstrate research hotspots and trends in pediatric ophthalmopathy, high-quality, newly current mainstream publications from the WoSCC database need to be selected for data analysis. The search strategy included limiting the database, search terms, language, document type and publication date, and the specific search strategy was as follows: the database was selected from the WoSCC database, with topic = (“pediatric*” or “paediatric*” or “child*” or “infant*” or “adolescent*” or “neonate*” or “inborn”) and title = (“eye*” or “ophthalm*” or “retin*” or “cornea*” or “keratitis” or “uveal” or “uveitis” or “scleral” or “orbital” or “orbitopathy”or “glaucoma”), with a language restriction of English, an document type of article, and a publication date period from January 1, 2014 to December 31, 2023. The data was checked separately by two researchers. Further, secondary selection was performed based on document type and article content, Proccedings papers, review articles, book chapters, early eccess, editorial materials, letters, meeting abstracts, corrections, data papers and retracted publications were excluded on the basis of document type. The content of publications matching the doucument type was then screened based on the each title and abstract. If unsure, the full text was downloaded and excluded based on content. Publications that were not relevant to the topic of pediatric ophthalmopathy were excluded, such as pediatrics alone or eye disease alone. Bibliometric and visualized analysis was performed on the included publications, and Figure 1 shows the detailed flowchart. In the data analysis, CiteSpace.6.2.6 was used to analyze the collaborative network of countries or regions, institutions, journals, as well as keywords and research categories, and the analysis function of the WoSCC database was used to analyze the number of annual publications and the number of national or regional publications. At the same time, high-impact publications were analyzed in depth and detail to further demonstrate the popular research on children's eye diseases.

## 3 Results

### 3.1 Annual number of publications

**Figure 2:**
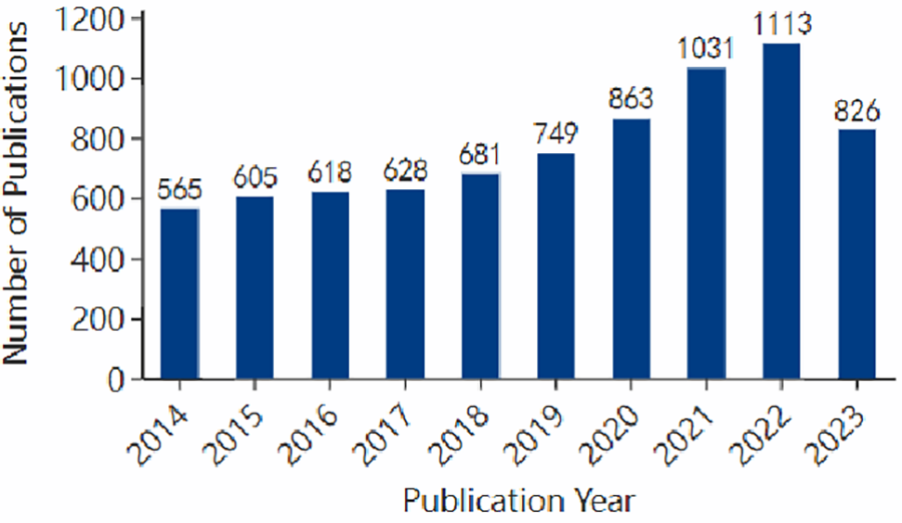
Annual number of publications on pediatric ophthalmopathy.

Following the identification and selection process, this study encompassed 7,177 publications specifically addressing pediatric ophthalmopathy. Over the past decade, the annual publication count had consistently exceeded 500, with a notable surge in recent years, peaking at over 1,000 in 2021 and 2022. While the number of publications for 2023 was slightly lower than 2022 due to some articles not yet being publicly available online at the time of data collection. The trend in annual publication counts for pediatric ophthalmopathy over the last decade is illustrated in Figure 2.

### 3.2 Countries or regions

**Figure 3:**
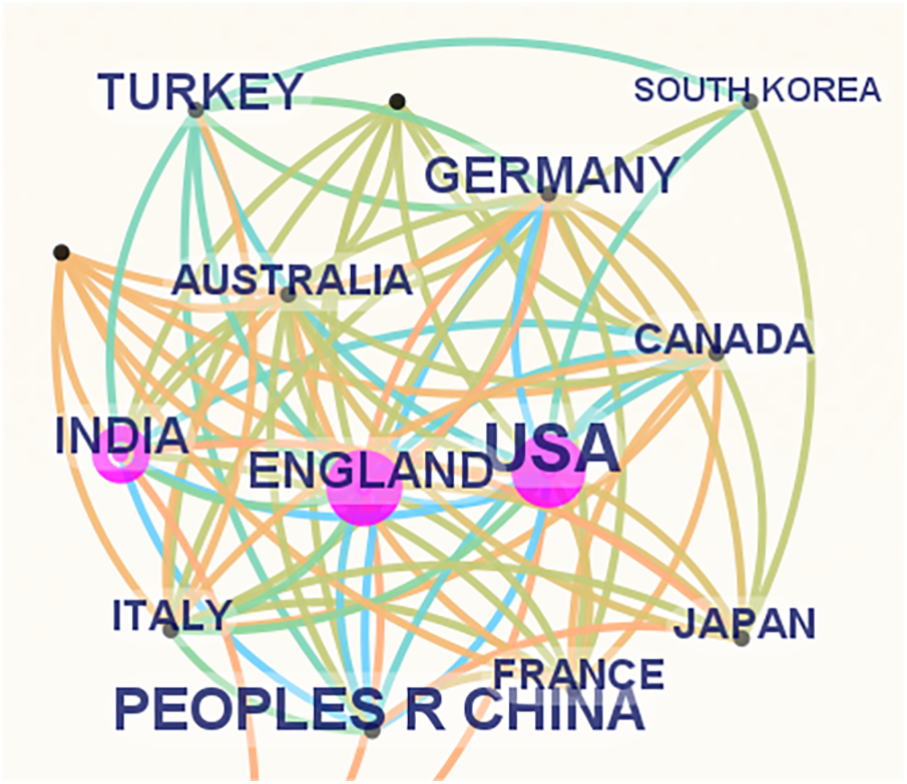
Cooperation of countries or regions that contributed to publications on pediatric ophthalmopathy.

These publications originate from 162 countries and regions. Figure 3 presents a collaborative network graph for each country or region, with the default settings of CiteSpace. The size of each label and node region in Figure 3 is proportional to the number of publications. The United States (2,269), the People's Republic of China (1,298), and India (649) had the largest labels and node regions, indicating the highest publication volumes. Connections between nodes signify cooperative efforts between countries or regions, with more connections indicating closer collaboration. Table 1 provides details for the top 10 countries or regions based on publication numbers, including centrality scores that reflect the strength of their cooperation and H-indexes that measure influence. The United States lead with the highest centrality (0.27) and H-index (66); China followed with a lower centrality but maintains a significant presence; England, with 561 publications, had a high centrality of 0.19 and an H-index of 40.

**Table 1:** Top 10 countries or regions with publications on pediatric ophthalmopathy.

Rank	Countries or regions	Counts	Centrality	H-index
1	United States	2,269	0.27	66
2	China	1,298	0.01	43
3	India	649	0.09	29
4	England	561	0.19	40
5	Turkey	458	0.00	27
6	Germany	371	0.08	35
7	Canada	329	0.04	35
8	Italy	313	0.03	29
9	Australia	267	0.07	32
10	Japan	238	0.00	25

### 3.3 Institutions

**Figure 4:**
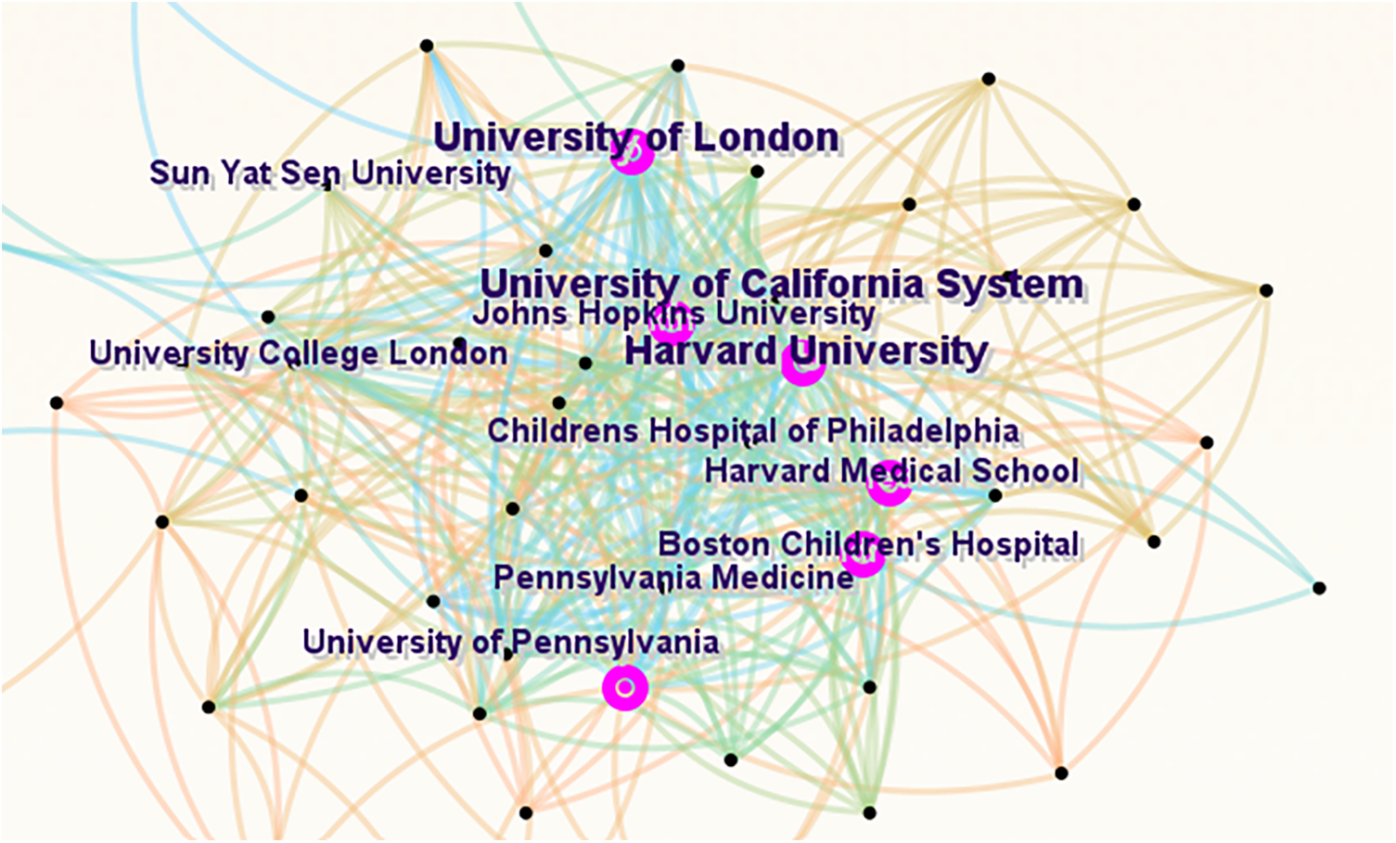
Cooperation of institutions that contributed to publications on pediatric ophthalmopathy.

Figure 4 displays the collaborative network graph for institutions, with the default settings of CiteSpace. Each institution's label and node represent the number of publications, and the connections between nodes indicate cooperative relationships. Table 2 ranks the top 10 institutions by publication volume. The University of London, Harvard University, and the University of California System were the top three institutions in terms of publication output. The University of London and Harvard University shared the highest H-index of 37; all institutions in the top 7 had an H-index of 30 or higher. The top 10 institutions included six from the United States, three from England, and one from Egypt.

**Table 2:** Top 10 institutions with publications on pediatric ophthalmopathy.

Rank	Institutions	Countries or regions	Counts	H-index
1	University of London	England	289	37
2	Harvard University	United States	265	37
3	University of California System	United States	257	31
4	University College London	England	215	34
5	University of Pennsylvania	United States	202	31
6	Pennsylvania Medicine	United States	183	31
7	Harvard Medical School	United States	179	31
8	Children's Hospital of Philadelphia	United States	158	28
9	Egyptian Knowledge Bank (EKB)	Egypt	140	19
10	Moorfields Eye Hospotal NHS Foundation Trust	England	139	27

### 3.4 Journals and research categories

Publications exhibit a citing-cited relationship, as do the journals themselves, with citing journals reflecting current research frontiers and cited journals representing the research foundation. Tables 3, 4 list the top 10 citing and cited journals, including journal names, research categories, citation counts, and the 2022 Journal Impact Factor. CiteSpace was utilized to visualize and analyze these relationships under default settings. The most prominent citing-cited relationship in research categories was neurology/sports/ophthalmology citing molecular/biology/genetics. The prevalent research categories in the top 10 citing and cited journals were ophthalmology, pediatrics, and science and technology.

**Table 3:** Top 10 citing journals of publications on pediatric ophthalmopathy.

Rank	Citing journals	Research categories	Counts	Journal impact factor 2022
1	Journal of AAPOS	Ophthalmology; Pediatrics	365	1.6
2	Indian Journal of Ophthalmology	Ophthalmology	258	3.1
3	PLoS One	Science & Technology	181	3.7
4	BMC Ophthalmology	Ophthalmology	163	2.0
5	Investigative Ophthalmology &Visual Science	Ophthalmology	159	4.4
6	American Journal of Ophthalmology	Ophthalmology	157	4.2
7	Scientific Reports	Science & Technology	153	4.6
8	Journal of Pediatric Ophthalmology &Strabismus	Ophthalmology; Pediatrics	151	1.2
9	Acta Ophthalmologica	Ophthalmology	143	3.4
10	European Journal of Ophthalmology	Ophthalmology	134	1.7

**Table 4:** Top 10 cited journals of publications on pediatric ophthalmopathy.

Rank	Cited journals	Research categories	Counts	Journal impact factor 2022
1	Ophthalmology	Ophthalmology	3,933	13.7
2	British Journal of Ophthalmology	Ophthalmology	3,512	4.1
3	American Journal of Ophthalmology	Ophthalmology	3,162	4.2
4	Investigative Ophthalmology & Visual Science	Ophthalmology	2,855	4.4
5	Journal of Aapos	Ophthalmology; Pediatrics	2,198	1.6
6	PLoS One	Science & Technology	2,076	3.7
7	Eye	Ophthalmology	1,926	3.9
8	Pediatrics	Pediatrics	1,758	8.0
9	Acta Ophthalmologica	Ophthalmology	1,449	3.4
10	JAMA Ophthalmology	Ophthalmology	1,358	8.1

### 3.5 Authors

**Figure 5:**
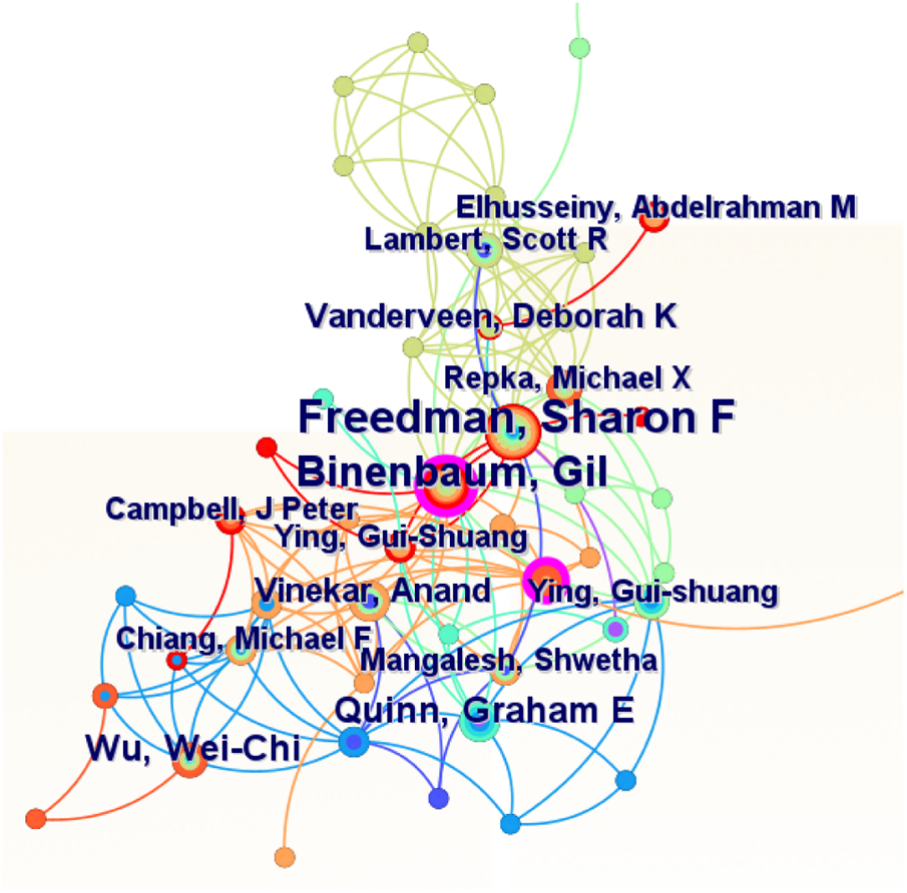
Cooperation of authors of publications on pediatric ophthalmopathy.

Figure 5 depicts the authors of publications and collaborations in the field of pediatric ophthalmopathy, with the node area indicating the volume of publications, the pink outer circle of the node denoting the influence, and the connecting lines representing the collaborative network. Table 5 exhibits information of the top 10 authors in terms of the number of publications, split into countries based on their institutions, with seven authors from the United States. The results reveal that Freedman,Sharon F was the most influential author with the highest number of publications in the field of pediatric ophthalmopathy.

**Table 5:** Top 10 authors of publications on pediatric ophthalmopathy.

Rank	Authors	Counts	Centrality	H-index	Countries or regions
1	Freedman, Sharon F	55	0.01	19	United States
2	Binenbaum, Gil	42	0.01	16	United States
3	Hellstrom, Ann	21	0.09	12	Sweden
4	Quinn. Graham E	21	0.19	13	United States
5	Wu, Wei-Chi	21	0.00	11	China, Taiwan
6	Vanderveen, Deborah K	18	0.08	11	United States
7	Vinekar. Anand	16	0.02	9	India
8	Lambert, Scott R	15	0.02	11	United States
9	Shields, Carol L	15	0.00	7	United States
10	Ying, Gui-shuang	15	0.01	8	United States

### 3.6 Keywords

**Figure 6:**
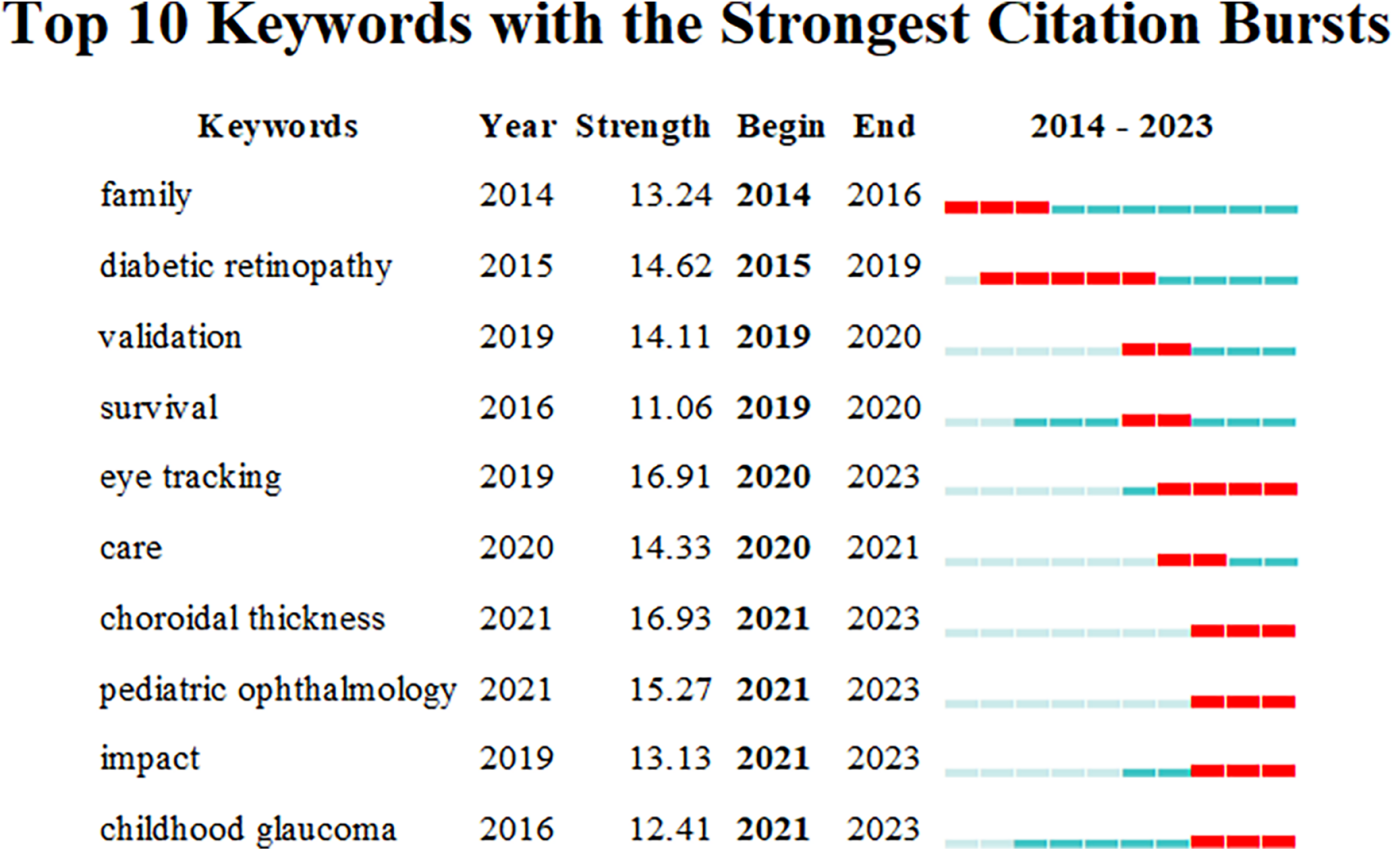
Keywords with the strongest citation bursts for publications on pediatric ophthalmopathy.

CiteSpace was employed to analyze co-occurring collaborative networks of keywords using the parameters: “Year Per Slice” = 1, “Top N%” = 10.0%, and “Minimum Duration” = 1. Figure 6 highlights the top 10 keywords with the strongest citation bursts, where strength indicates the intensity of the keyword's emergence. The red squares denote the timeline of keyword surges. “Diabetic retinopathy” and “eye tracking” were keywords with the longest periods of activity, while “eye tracking,” “choroidal thickness,” “pediatric ophthalmology,” “impact,” and “childhood glaucoma” were keywords that have emerged within the last three years.

### 3.7 High-impact publications

High-impact publications with high cited counts are indicative of well-regarded findings in pediatric ophthalmopathy, demonstrating the research hotspots in this field. Table 6 lists the top 10 most highly cited publications in this field, covering aspects of pathogenesis, diagnosis, and treatment of ocular diseases in children. These articles predominantly featured clinical studies, particularly those focused on the treatment of retinopathy of prematurity, alongside mechanistic studies such as one exploring the metastatic pathway of retinoblastoma. The types of diseases were related to retinopathy of prematurity (4), myopia (2), retinoblastoma (1), uveitis in juvenile idiopathic arthritis (1), and ocular manifestations of systemic diseases (2).

**Table 6:** Publications with top ten citation on pediatric ophthalmopathy.

Rank	Title of publication	DOI	Times cited	Interpretation of the findings
1	Low-concentration atropine for myopia progression (LAMP) study A randomized, double-blinded, placebo-controlled trial of 0.05%, 0.025%, and 0.01% atropine eye drops in myopia control (65)	10.1016/j.ophtha.2018.05.029	298	0.05%, 0.025%, and 0.01% atropine eye drops slowed myopic progression in a concentration-dependent response. And 0.05% atropine was most effective in controlling spherical equivalent progression and axial length elongation over 1 year.
2	Adalimumab plus methotrexate for uveitis in juvenile idiopathic arthritis (66)	10.1056/NEJMoa1614160	244	For uveitis in juvenile idiopathic arthritis, adalimumab in combination with methotrexate had a lower failure rate than placebo, but a higher rate of adverse events than placebo.
3	Neurodevelopmental outcomes following bevacizumab injections for retinopathy of prematurity (67)	10.1542/peds.2015-3218	206	Bevacizumab-treated versus laser-treated preterm infants with retinopathy have a higher incidence of severe neurodevelopmental disorders.
4	Ophthalmological findings in infants with microcephaly and presumable intra-uterus Zika virus infection (68)	10.5935/0004-2749.20160002	204	Infants with microcephalyhad a normal anterior segment and severe abnormalities in the macula and optic nerve.
5	Ranibizumab versus laser therapy for the treatment of very low birthweight infants with retinopathy of prematurity (RAINBOW): an open-label randomised controlled trial (69)	10.1016/S0140-6736(19)31344-3	198	For the treatment of retinopathy of prematurity, ranibizumab 0.2 mg may be superior to laser therapy with fewer adverse local outcomes.
6	Eye tracking reveals abnormal visual preference for geometric images as an early biomarker of an autism spectrum disorder subtype associated with increased symptom severity (70)	10.1016/j.biopsych.2015.03.032	189	Enhanced visual preference for geometric repetition may be an early developmental biomarker of an ASD subtype with more severe symptoms.
7	Inhibition of MMP-2 and MMP-9 decreases cellular migration, and angiogenesis in in vitro models of retinoblastoma (71)	10.1186/s12885-017-3418-y	188	MMP-2 and MMP-9 drived the metastatic pathways of retinoblastoma, including migration, viability, and secretion of angiogenesis.
8	A dosing study of bevacizumab for retinopathy of prematurity late recurrences and additional treatments (72)	10.1016/j.ophtha.2018.05.001	178	Low-dose bevacizumab for retinopathy of prematurity had a very favorable outcome, but many eyes received other treatments.
9	Outcomes after intravitreal bevacizumab versus laser photocoagulation for retinopathy of prematurity a 5-year retrospective analysis (73)	10.1016/j.ophtha.2014.12.017	153	Intravitreal bevacizumab versus panretinal photocoagulation is an effective treatment option for retinopathy of type 1 prematurity, which has a low rate of myopia.
10	Effect of outdoor activity on myopia onset and progression in school-aged children in Northeast China: the Sujiatun eye care study (74)	10.1186/s12886-015-0052-9	151	Increased outdoor activity prevents the onset and progression of myopia in children, as well as axial growth and increased intraocular pressure.

## 4 Discussion

### 4.1 Overall data

Pediatric ophthalmopathy directly impair children's vision, affecting not only their health and quality of life but also their families and society, making pediatric ophthalmopathy a focal point for research. This study analyzed publications in the past decade from the WoSCC database to obtain research hotspots and trends in the field of pediatric ophthalmopathy. The analysis indicates that over the past decade, there has been a significant increase in pediatric ophthalmopathy research, particularly after 2021, with an annual publication count exceeding 1,000. In terms of national or regional contributions, the United States lead with the highest number of publications, centrality, and H-index, reflecting its dominant role in the field. China, ranking second in publication volume and H-index but with lower centrality, also exert significant influence, suggesting a need for enhanced international collaboration. England, despite having fewer publications, demonstrated high centrality and influence, indicating the value and collaborative nature of its research. Institutions such as the University of London and Harvard University stand out with the highest H-index, indicating their substantial impact. Freedman,Sharon F was the most authoritative author, and most of the authors with high posting volume and influence were from the United States. The journal research categories revealed a focus on pediatrics, ophthalmology, and science and technology, aiming to develop more effective diagnostic and treatment methods for pediatric ophthalmopathy. The analysis of burst keywords highlights the current research hotspots, and highly cited articles showcase impactful research findings, with retinopathy of prematurity, myopia, and retinoblastoma emerging as prominent areas of study. Specifically, four articles on retinopathy of prematurity were clinical studies assessing the efficacy and adverse effects of various treatments, including laser therapy, bevacizumab, and ranibizumab. These treatments showed potential clinical efficacy, but bevacizumab, when compared to laser therapy, was associated with a higher incidence of severe neurodevelopmental disorders, while ranibizumab therapy was found to be superior, with fewer adverse outcomes.

### 4.2 Research hotspots

The analysis of the publications on pediatric ophthalmopathy in the WoSCC database over the past 10 years provides access to the research hotspots in this field. Emerging burst keywords responds to the research hotspots. The evolving burst keywords across different time periods reflect the shifting research focus in pediatric ophthalmopathy. In 2014–2016, the keyword “family” emerged, primarily encompassing family-related and genetically linked congenital eye diseases (18–20), as well as aspects of family care (21, 22). The keyword “diabetic retinopathy” gained prominence from 2015 to 2019, with a focus on early detection markers before clinical symptom onset, including retinal microcirculation changes (23), retinal nerve fiber layer thickness (24), choroidal thickness (25), and the integration of artificial intelligence methods (26). The keywords “validation” and “survival” emerged in 2019–2020, with “validation” referring to the clinical validation of guidelines, standards, and questionnaires related to pediatric ophthalmopathy (27, 28), and the capabilities of artificial intelligence platforms (29, 30). Additionally, “care” became a significant keyword in 2020–2021, highlighting the crucial role of care in pediatric ophthalmopathy (31, 32). The keywords “eye tracking” and “choroidal thickness” emerged in 2020–2023, followed by “pediatric ophthalmology,” “impact,” and “childhood glaucoma” in 2021–2023, indicating the latest research trends in the field.

“Eye tracking” technology, which can automatically monitor the visual dynamics of young children, has been applied in screening for autism spectrum disorder (33, 34), detecting glaucoma (35), quantifying the severity of intermittent esotropia (36), and assessing vision in infants and children (37). Ahmed et al. developed three AI techniques for early autism diagnosis using eye-tracking data, all achieving over 90% accuracy (38). Wen et al. proposed an automated acuity card procedure based on eye-tracking technology for assessing children's visual acuity, with results comparable to Teller Acuity Cards (37).

“Choroidal thickness” emerged as a keyword with significant intensity, serving as a critical indicator for assessing myopia development in children (39, 40). Zhu et al. found a positive correlation between the degree of myopia in children and choroidal thickness using Pearson correlation analysis (41). Jiang et al. discovered that choroidal thickness thinning in myopic patients was more pronounced nasally than temporally and superiorly, suggesting that asymmetric nasal predominance of thinning choroidal thickness could be a new biomarker for early-onset high myopia (42). Monitoring choroidal thickness may thus provide valuable insights, aligning with the high citation of articles focusing on myopia as the predominant disorder.

“Pediatric ophthalmology,” the dominant burst keyword from 2021 to 2023, signifies a growing interest in the field as a whole, with an increasing number of studies focusing on pediatric ophthalmology in general (43), rather than solely on children, pediatrics, or specific diseases. Ramsay et al. conducted a retrospective analysis of the epidemiology and diagnosis of pediatric ocular diseases in ophthalmology emergencies, summarizing patterns of pediatric ophthalmology consultations in such situations (44). As a subspecialty, this keyword also encompasses the development of pediatric ophthalmology, its economic and social impact (45–47), and the impact of economic factors, such as recessions, on the field and eye care (48).

The keyword “impact” addresses the effects of treatments and social factors on pediatric ophthalmopathy, as well as the repercussions of these conditions on family life and psychological well-being. Luccarelli et al. investigated the efficacy of oral acyclovir in preventing herpetic keratitis recurrences, demonstrating its safety and effectiveness (49). Shah et al. explored the impact of COVID-19 on pediatric ophthalmology emergency services, noting a decline in attendance (50). da Silva et al. assessed the psychosocial implications of surgical treatment for primary congenital glaucoma on children and their families (51).

“Childhood glaucoma,” a prevalent research topic, is characterized by a persistent increase in intraocular pressure, potentially leading to blindness. Surgical treatments for childhood glaucoma are a significant research area, encompassing procedures such as trabeculotomy (52), combined trabeculotomy-trabeculectomy (53), angio-atriotomy (54), Ahmed glaucoma drainage device implantation (55, 56), Baerveldt glaucoma drainage device implantation (57), ciliary body photocoagulation (58), and XEN gel stent implantation (59), as well as penetrating otoplasty for refractory cases (60). Visual outcomes have been correlated with factors such as age at diagnosis, corneal clouding, and the presence of concurrent amblyopia (61, 62). Recent research has also identified associations with genetic mutations (63, 64).

### 4.3 Trends discussion

The increasing number of publications in pediatric ophthalmopathy represents that this field is in a research hotspot. The emerging burst keywords and high-impact publications in recent years represent the changing trends in the field of pediatric ophthalmopathy. Myopia, childhood glaucoma, retinopathy of prematurity, retinoblastoma and uveitis in juvenile idiopathic arthritis are the hot diseases in research, and modern technology such as eye tracking has become a popular application technology for the diagnosis and intervention of pediatric ophthalmopathy, and the current research has focused more on the overall relationship between pediatric ophthalmopathy and social factors. These hot diseases, hot technologies, and holistic research are the research trends in the field of pediatric ophthalmopathy, which can inform clinical practice and will also become the directions of future research, and further improve eye health in children.

### 4.4 Limitations and resolvents

This study acknowledges several limitations that may affect the comprehensiveness and generalizability of the findings. Firstly, in order to high-quality articles, the publication selection was constrained by the use of the Web of Science Core Collection (WoSCC) database exclusively, which may have resulted in an incomplete collection of relevant literature. Utilizing multiple databases could have provided a more comprehensive view. Secondly, the search was restricted to English-language articles, potentially excluding valuable research from other linguistic domains. Thirdly, the document type was also limited to article, which may have omitted important contributions from other formats such as conference proceedings or reviews. And, some research results were not published in publications. And time constraints have been imposed to obtain hotspots for new timelines, which may lead to incomplete literature coverage. Additionally, the research content of the selected publications presents certain limitations. In clinical studies on medications, treatments, and pathogenesis of pediatric ophthalmopathy, the sample sizes are small, necessitating larger samples and cross-regional studies for more robust findings. The application of artificial intelligence in pediatric ophthalmopathy, while a growing area of scientific research, lacks sufficient clinical application studies and consistent models, which could hinder its practical implementation. The integration of resources to facilitate clinical research in this area is needed. Furthermore, the understanding of the underlying mechanisms of pediatric ophthalmopathy remains limited, indicating a need for more in-depth research in this domain.

## 5 Conclusion

This study offers a systematic bibliometric and visualized analysis of pediatric ophthalmopathy research over the past decade, providing a comprehensive overview of the field's current status and research hotspots. The study reveals a growing global interest in pediatric ophthalmopathy, particularly post-2021, with the United States and China exerting the most significant influence. Prominent institutions include the University of London and Stanford University, and Freedman,Sharon F was the most prominent. The primary research categories encompass pediatrics, ophthalmology, and science and technology, with a focus on diseases such as diabetic retinopathy, childhood glaucoma, retinopathy of prematurity, and myopia. Key areas of interest include “eye tracking,” “choroidal thickness,” “pediatric ophthalmology,” “impact,” and “childhood glaucoma.”

In summary, the study highlights ongoing in-depth research into the pathogenesis, screening, prevention, and treatment of pediatric ophthalmopathy. The integration of modern science and technology, such as eye tracking and deep learning, may offer more reliable methods for assessing and diagnosing these conditions. Further investigation into choroidal thickness could yield novel biological markers for myopia severity. Additionally, there is a growing emphasis on understanding the impact of childhood eye diseases on families and society. Given that ocular abnormalities in children can lead to irreversible vision damage and adverse effects on children eye health, early diagnosis and treatment are crucial for the prognosis of children's vision. Current research in pediatric ophthalmopathy primarily focuses on etiology, diagnosis, and treatment. However, there is a need for extensive research into the genetics and molecular mechanisms of pediatric ophthalmopathy to provide a more comprehensive and effective foundation for clinical practice.

## Data Availability

Publicly available datasets were analyzed in this study. This data can be found here: https://www.webofscience.com/.
